# Development of a carbon paper-based electrochemical immunosensor for trimethoprim detection in fishery species: monitoring antibiotic contaminants

**DOI:** 10.5599/admet.2962

**Published:** 2025-10-06

**Authors:** Maria Freitas, Vitória Dibo, Rita Ribeiro, Cristina Delerue-Matos, Simone Morais, Álvaro Torrinha

**Affiliations:** REQUIMTE/LAQV, ISEP, Polytechnic of Porto, Rua Dr. António Bernardino de Almeida, 431, 4249-015, Porto, Portugal

**Keywords:** Amperometry, biosensor, paper-based electrode, pharmaceuticals, drug analysis, aquatic ecosystem

## Abstract

**Background and purpose:**

Monitoring antibiotic drugs in the environment is particularly relevant given their role in fostering microbial resistance, impacting aquatic species and human health. Therefore, this work addresses the lack of a sustainable and cost-effective analytical approach and reports the development of a competitive electrochemical immunosensor for the rapid analysis of trimethoprim (TMP), an aquatic contaminant of emerging concern, overcoming the limitations of conventional methods that are often costly and time-consuming.

**Experimental approach:**

A miniaturized 3-electrode system was developed to support the analyses of a low-volume sample (200 μL), using a low-cost carbon-paper working electrode, pencil graphite lead auxiliary electrode and a stainless-steel needle reference electrode. Scanning electron microscopy was used to characterize the transducer, revealing a network of randomly arranged carbon fibres, significant for efficient antibody immobilization. For the immunoassay construction, an anti-TMP antibody was physically adsorbed to a small-sized transducer (*d* = 4 mm) for the specific recognition of TMP, followed by incubation with the enzyme-conjugate (horseradish peroxidase) and the enzyme-substrate (tetramethylbenzidine).

**Key results:**

The analytical signal was recorded by chronoamperometry (1-minute reaction) and yielded a limit of detection of 34 ng L^−1^. The 45-minute assay demonstrates accuracy (92.0 to 103.2 % in fishery species including codfish, mackerel, crab, and hake), reproducibility (6.1 and 9.3 % for repeatability and inter-day variation coefficient) and high selectivity (bias less than 5 %) analysis. The sensor's performance was validated against a conventional Enzyme-Linked Immunosorbent Assay.

**Conclusion:**

This study introduces a sustainable electrochemical immunosensor, offering a portable and eco-friendly alternative for the rapid detection of TMP in fishery species, addressing the limitations of traditional methods.

## Introduction

Monitoring contaminants of emerging concern (CEC) using high-throughput analytical techniques is critical to lay down regulations and preventive measures to protect aquatic ecosystems and human health [1]. Pharmaceuticals are essential commodities for modern society, ensuring well-being and growth in life expectancy, but they also raise environmental concerns due to their increasing prevalence, potential persistence, bioaccumulation and toxic properties. Thus, they are considered a relevant CEC class due to their potential toxicity and bioaccumulation capacity that affect aquatic ecosystems and human health. Due to negligent disposal and inefficient wastewater treatment, these compounds are widely found in environmental waters [2-4].

Antibiotic quantification in environmental matrices is particularly relevant due to the potential risk of microbial resistance [5]. Specifically, trimethoprim (TMP) is a widely prescribed antibiotic drug for human care and, therefore, can be largely detected in aquatic matrices. Additionally, TMP is commonly applied in aquaculture, and concerns about its persistence and potential contamination of fishery species have been raised. In this concern, the European Commission established a maximum residue limit (MRL) for TMP in the tissues of food-producing species of 50 μg kg^−1^ [6]. Also, the European Union's Drinking Water Directive (Directive (EU) 2020/2184) [7] stated that if TMP is detected as a contaminant, its presence could be subject to general limits imposed for pesticides (0.1 μg L^−1^ for individual pesticides). Excessive antibiotic residues in aquatic environments can pose risks to human health, contribute to antibiotic resistance, and affect ecosystem balance. However, the chromatographic and enzymatic current analytical detection methods to detect TMP in fishery products are costly, labor-intensive, depend on advanced instrumentation and involve specialized facilities, limiting their practical application for routine monitoring [8-10].

The development of analytical solutions to determine this drug has recently gathered attention from the scientific community due to its inclusion in the European Commission Watch List for surveillance of potentially hazardous substances present in surface waters [11]. Several studies have confirmed the presence of TMP in aquatic matrices, mainly based on high-performance liquid chromatography (HPLC), which, despite being reliable and multiplexed, are still bulk and confined to laboratory facilities, requiring specialised personnel to operate. Enzyme-linked immunosorbent Assay (ELISA) can also be applied; however, it is a time-consuming technique and requires expensive equipment [12-14]. Still, additional improvements are needed to enhance efficiency by increasing sensitivity and selectivity and reducing fabrication costs. Unlike conventional detection techniques, (bio)sensors offer a portable alternative for real-time monitoring, emerging as a powerful analytical tool to detect contaminants [15].

Electrochemical sensors provide accurate, selective and rapid detection of antibiotic residues. TMP analysis was widely performed using conventional glassy carbon electrodes (GCE) [16-20] and carbon paste electrodes (CPE) [21-26]. However, small-sized and sustainable materials offer significant advantages for current regulations and practices related to TMP monitoring. Its ease of use allows rapid on-site testing, potentially shifting the paradigm from centralized laboratory analysis to decentralized, field-based monitoring, thereby influencing current testing processes to improve practices and regulations [27-29]. Other miniaturized alternatives included a molecularly imprinted interface constructed on an acupuncture needle, but the manufacturing process required significant amounts of high-purity reagents and specific materials to achieve the functional surface and specific nanocavities required for selective TMP detection [30]. Nonetheless, the integration of a bioreceptor can improve the selectivity of the biosensing strategy.

Based on the literature review, no biosensing strategy or immunosensor has been developed to analyse and quantify TMP in fishery products. To address this issue, the development of an electrochemical immunosensor using recyclable-based (cell) and carbon paper-based material presents a promising alternative. The challenge lies in optimizing this miniaturized sensor to ensure its reliability and real-world applicability for effective monitoring in the aquaculture industry [1]. Thus, in this work, an antibody was applied as a biorecognition element (anti-TMP antibody) and immobilized onto a sustainable transducer (carbon paper electrode) for the specific determination of TMP (analyte) in fishery species, addressing the growing demand for efficient and eco-friendly antibiotic monitoring solutions in the aquaculture industry. The electrochemical immunosensor was constructed in a competitive assay format and applied to quantify the selected drug in several fishery species. In this assay, the analyte was recognized by an antibody labelled with a horseradish enzyme (HRP) and the product of the enzymatic catalysis was processed through a reduction process after the addition of the enzymatic substrate tetramethylbenzidine (TMB), measured by chronoamperometry. Simple and widely available electrochemical materials, such as carbon paper, pencil graphite lead, and a stainless-steel needle, were used simultaneously for the first time in the assembly of a three-electrode cell, aiming to further reduce the cost and miniaturize the electrochemical cell for low sample volume analysis. The use of sustainable materials enhances the environmental friendliness of this device, making it suitable for widespread application in food safety and environmental monitoring; however, it poses a potential challenge in real-world applications.

## Experimental

### Equipment

Electrochemical measurements were carried out using a potentiostat/galvanostat (PGSTAT101) with NOVA software (v.1.10) (Metrohm Autolab, The Netherlands). Chronoamperometry measurements were performed by using carbon paper (Toray TGP-H-60, 0.56×2.0 cn, Alfa Aesar, Germany) as the working electrode (WE, *d* = 4 mm), pencil graphite lead (*d* = 1 mm, Rotring, Germany) as the auxiliary electrode (AE), and stainless-steel needle (Neolus, Belgium) as the reference electrode (RE). The 3-electrode system was immersed in an electrochemical microcell (1.0×1.0 cm) to perform the measurements. For food sample preparation, a vortex (VWR, Germany) and a centrifuge (Heraeus Megafuge 16R, Thermo Fisher Scientific, Germany) were used. Scanning electron microscopy (SEM) was performed at the “Centro de Materiais da Universidade do Porto (CEMUP)”, using FEI Quanta 400FEG ESEM/EDAX Genesis X4 M equipment (USA).

### Reagents and solutions

N-hexane and methanol were purchased from Carlo Erba. Ascorbic acid (Riedel-de Haen), sulfamethoxazole, and sulfadiazine (Sigma-Aldrich) were applied as interference substances. Trimethoprim (>99 %), polyclonal anti-trimethoprim antibody (anti-TMP), horseradish peroxidase-conjugated to antibody (HRP-conjugate), 3,3′,5,5′-tetramethylbenzidine (TMB) with hydrogen peroxide (H_2_O_2_), phosphate buffer 20 mM with sodium chloride 150 mM, pH 7.4 (PBS), redissolving solution (PBS with BSA 5 %), and an Enzyme-Linked Immunosorbent Assay kit (ELISA, MBS286552) were acquired from MyBioSource (USA). The PBS buffer solution was used for diluting the biomolecules and served as a supporting electrolyte.

### Preparation of the 3-electrode microsystem

**Figure 1.**
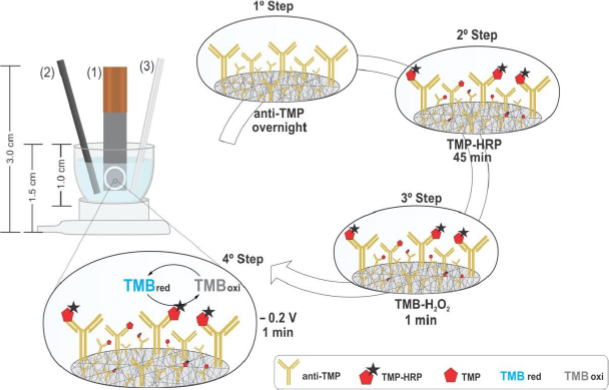
Schematic representation of the homemade sustainable electrochemical system: (1) carbon paper- WE; (2) pencil graphite lead - AE; (3) stained steel needle - RE. The electrochemical measurements were performed inside a microcell with a 200-μL electrolyte solution - a representation of the competitive immunosensor construction for the analysis of trimethoprim

The developed miniaturised electrochemical system is presented in Figure 1 and consists of 3 sustainable electrodes: (1) carbon paper (WE), with a circular Teflon mask added to delimit the working area (*d* = 4 mm), and a conductive copper tape, used for electrical contact; (2) pencil graphite lead (AE, *d* = 1 mm), and (3) stainless-steel needle (RE). A homemade microcell (dimensions = 1.0×1.0 cm) allowed contact with the electrolyte solution (volume = 200 μL).

### Immunosensor construction

The schematic representation of the biosensor construction comprised the following steps: (1) the biomodification of the delimited WE, with anti-TMP (10 μL, 5 μg mL^-1^, incubated overnight in a moist chamber). Subsequently, excess solution was removed, the electrode surface was washed with PBS buffer and the competitive immunoassay protocol included the following steps: (2) simultaneous addition of TMP standard and sample (5 μL), and HRP-conjugate (enzyme conjugate, 5 μL without dilution), incubated for 45 min; removal of the excess solution, wash with PBS buffer to guarantee the removal of non-specifically adsorbed biomolecules and (3) the WE was subsequently immersed (for 1 min) into the microcell which contained the electrolyte solution (enzymatic substrate TMB-H_2_O_2_, 200 μL). The chronoamperometric measurement (4) was recorded according to [31], applying a constant potential of -0.2 V and obtaining the current associated with the reduction process onto the WE. The detection mechanism involved the electrochemical oxidation of the enzyme conjugate (HRP-conjugate) with the enzymatic substrate (TMB-H_2_O_2_) and the subsequent reduction process on the WE surface, thereby generating the analytical signal. Three replicates were carried out for each analysis.

### Food samples preparation

Fish samples (codfish, horse mackerel, white crab, hake) were purchased at local supermarkets (Portugal). Each sample was homogenized, and 2 g of the tissue was mixed with 6 mL methanol and 2 mL n-hexane, shaken for 5 min, and centrifuged (4000 rpm, 20 °C, 10 min). The upper layer (organic phase) was removed, and an aliquot of the remaining layer (500 μL) was collected into a microtube and dried under a nitrogen flow. The residue was dissolved with the redissolving solution (400 μL) and N-hexane (500 μL). After an additional centrifugal step and the supernatant removal, 50 μL of the remaining layer was collected and stored (4 °C, for 1 week) for analysis (fold of dilution: 5×). The obtained samples were analysed with the constructed immunosensor. The biosensor results were compared with those reached using a commercial ELISA kit (kit MBS286552, competitive assay procedure, MyBioSource, USA) and a multi-mode microplate reader (Synergy HT W/TRF, BioTek Instruments, USA).

## Results and discussion

### Electrochemical microsystem construction and optimization

**Figure 2.**
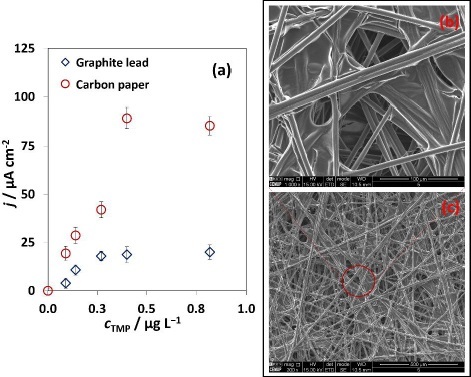
(a) Selection of the working electrode (WE) for the electrochemical system construction: graphite lead (WE), and carbon paper (WE), both cells using a stainless-steel needle as the reference electrode and graphite lead as the auxiliary electrode. Electrochemical measurements were recorded by linear sweep voltammetry (0.8 to 1.4 V, sweep scan rate 0.05 V s^-1^); (b) and (c) scanning electron micrograph of the carbon paper electrode (bar scale: 100 and 500 μm, respectively)

The miniaturized electrochemical system consisted of a 3-electrode system constructed from recyclable and reusable materials, immersed in a homemade microcell. A plastic microcell with reduced dimensions was designed for a total electrolyte volume of 200 μL. To explore environmentally sustainable electrodes, different carbon-based working electrodes were tested and characterized by linear sweep voltammetry (LSV, 0.8 to 1.4 V, sweep speed 0.05 V s^-1^) for TMP signal in 20 mM PBS buffer (supporting electrolyte): graphite lead (*d* = 2 mm) and carbon paper (CP, *d* = 4 mm), both using a stainless-steel needle (*d* = 0.9 mm) as reference electrode and graphite lead (*d* = 1 mm) as the auxiliary electrode. The 3-electrode system was connected, and an initial test was carried out to choose the most suitable WE by adding increasing TMP concentrations (0.0, 0.03, 0.09, 0.14, 0.27, 0.40 and 0.81 μg L^-1^). The obtained signals (oxidation peak at around +1.17 V) were normalized by the circular geometric area (expressed in current density/area (*j* / μA cm^-2^), in which the calculated active surface area (0.126 cm^2^) was calculated according to the WE diameter. The CP presented significantly higher current densities than the graphite lead (Figure 2a), revealing that the CP was the preferred WE, which led to a considerably better electrochemical signal. This is attributed to the high porosity and surface area provided by the characteristics of the carbon paper material, which enables a higher loading capacity of biomolecules and electrocatalytic activity [32]. Regarding the tested TMP concentrations, the signal saturation was observed from 0.40 μg L^-1^ onwards, reflected by its stability.

Although this approach allowed the direct detection of TMP (label-free assay), it presents reduced sensitivity when analysing samples with a wide range of concentrations, also with poor selectivity due to the high potential of the oxidation peak. Therefore, the construction of a competitive-type immunosensor is a considerably advantageous alternative, alongside the practicable combination with enzyme catalysis for signal amplification [1]. Still, biomolecules are incubated onto the working electrode and thus the material constitution is essential for appropriate bonding. In this context, the carbon paper material was analysed through SEM and the micrograph revealed a network of randomly arranged carbon fibres (Figure 2b and 2c), significant for efficient antibody immobilization since this carbon material possesses feasible physical characteristics for the efficient biomolecule residue bonding. Physical adsorption is advantageous in this strategy, as it enables direct binding, making the process simpler and cheaper, and avoiding potential surface blockages caused by additional reagents, as in the case of covalent binding.

### Optimization of the experimental variables involved in the preparation of the immunosensor

A significant objective of this study was the development of a microcell to considerably reduce the biomolecule volume for assay performance (in a microliter level), compared to the conventional electrochemical system (usually in millilitre). Thus, the 3-electrode system was arranged in the microcell using a CP electrode of reduced dimensions, where the working area was previously delineated. The biosensor optimization comprised several experimental parameters, namely (a) biomolecule volume onto the WE surface, (b) enzyme conjugate dilution, (c) antibody concentration and (d) incubation time.

**Figure 3.**
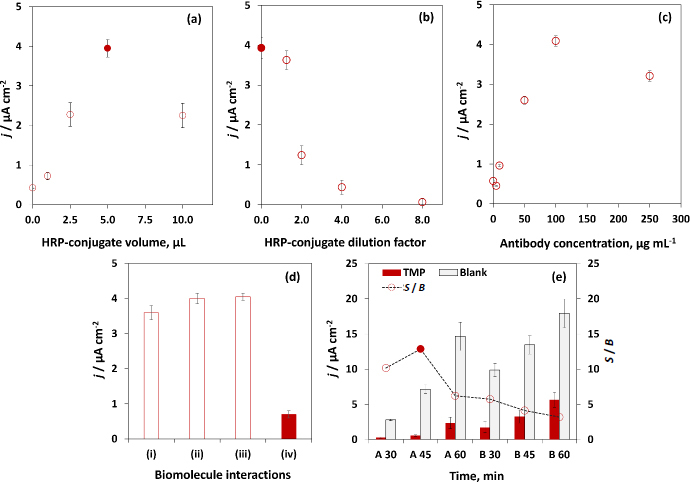
Optimization of experimental parameters: (a) volume of HRP-conjugate immobilized on the WE surface, (b) How many times HRP conjugate was diluted, and (c) concentration of anti-TMP antibody. (d) Control studies evaluating biomolecule interactions in the absence of antibody, target analyte, or enzyme-conjugate, and in the presence of all components. (e) Time assay formats: sequential addition of TMP and HRP-conjugate (*A*) *vs.* pre-incubation (B) pre-incubation of TMP+HRP-conjugate, 10 min before addition onto the WE surface

Testing the effective bond between the enzyme and the enzyme conjugate is essential to obtain the electrochemical signal. Therefore, the enzyme conjugate (HRP) was immobilized onto the WE surface, and the substrate (TMB-H_2_O_2_) was subsequently added. To optimize the aliquot volume to be immobilized on the WE surface, distinct volumes of the HRP-conjugate (0, 1, 2.5, 5, 10 μL, without dilution) were incubated for 45 minutes. As shown in Figure 3a, the signal intensity increases when immobilizing 5 μL of the biomolecule, which is selected to continue the optimization process and achieve the best analyte recognition efficiency, without exceeding the delimited WE area.

Subsequently, HRP-conjugate dilutions (0.0, 1.25, 2.0, 4.0 and 8.0 ×) were assessed to verify the optimal ratio for substrate conjugation, and electrochemical measurements were carried out after the enzymatic reaction. Regarding the enzyme-conjugate dilutions (carried out in PBS buffer), the results (Figure 3b) indicated that the higher dilutions led to a decrease in signal intensity, as expected. Additionally, for lower dilutions, the signal approaches saturation. The dilution of the conjugate affects the greater or lesser competition between TMP and labelled TMP for the binding sites of the immobilized antibody. Thus, the appropriate conjugate concentration should be selected to allow an adequate competition between TMP and TMP-HRP conjugate for the anti-TMP binding sites. Therefore, it is concluded that the HRP-conjugated dilution is not beneficial to the assay.

In the competitive immunoassay format, the selected antibody and its concentration play a crucial role in the sensitivity of target analyte detection, especially when similar drugs may exist in the sample, as they ensure the effective detection of the target analyte. Thus, the anti-TMP antibody was evaluated by immobilizing overnight onto the electrode surface in distinct concentrations (5.0, 10, 25, 50, 100 and 250 μg mL^-1^). Then, TPM was incubated (0.81 μg L^-1^) along with the HRP-conjugate for 45 min. After the reaction, the current increased with the concentration of antibody, indicating that the lower antibody loadings were not enough to bind the TMP with the HRP-conjugated. However, for the higher concentration of anti-TMP, the current decreased, suggesting the blockage of the electrode surface by the biomolecule. Thus, it is noticeable that the best result was observed for anti-TMP 100 μg mL^-1^ (Figure 3c), indicating that the higher signal corresponds to the effective antibody binding site's bioconjugation.

Additionally, biomolecule interactions were tested for the control analysis to assess the correct immunosensor configuration related to the appropriate biomolecule bonding (Figure 3d). A possible loss of functionality can occur (*e.g.* denaturation), leading to the absence of an analytical signal since all the assay constituents are required for a full assay performance [33]. Thus, the assay was tested in the absence of the main components, (i) anti-TMP antibody, (ii) TMP, (iii) HRP-conjugate, and (iv) in the presence of all the bioreagents. As observed, the correct signal acquisition is obtained in (iv), corresponding to the presence of the biomolecules, which corroborates the accurate results.

Furthermore, the incubation time required for the reaction between biomolecules to occur is essential for an efficient analysis of the target analyte. Short times may not guarantee effective binding, while long times may lead to cross-reactions (Figure 3e). Therefore, the following parameters were studied: (A) the typical direct addition of biomolecules to the WE surface, and (B) the pre-incubation step that can lead to signal improvement, resulting from the prior binding of the analyte with the enzyme conjugate (in a microtube, for 10 min) and subsequent addition to WE. Both strategies include time assay optimization for 30, 45 and 60 min. In this optimisation, TMP was tested at 0.0 μg L^-1^ (blank) and 0.81 μg L^-1^ (signal), and the signal-to-blank ratio (*S*/*B*, where *S* is the analytical signal obtained when the solution contains TMP and *B* is the analytical signal obtained in the absence of TPM) allowed the selection of the optimal result, which corresponds to the parameter *A* (direct addition), related to incubation for 45 min.

### Analytical performance

**Figure 4.**
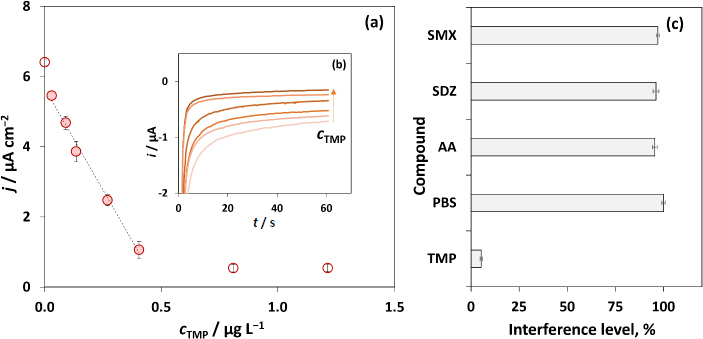
(a) Calibration plot (variation of the current density with the concentration of the standard TMP using the delimited carbon paper electrode, under the optimized conditions; (b) Representative chronoamperograms for different TMP concentrations. (c) Interference analysis performed in mackerel samples in the presence of TMP and sulfamethoxazole, sulfadiazine and ascorbic acid, in a concentration of 0.40 μg L^−1^ and the absence of any pharmaceutical compound

Detection of low drug concentrations under evaluation requires a signal enhancement that can be recorded through an enzymatic reaction in a competitive-type assay format. Thus, the biosensor analytical characteristics were assessed using the optimized experimental variables. To establish the working range, the relationship between the electrochemical signal (*j* / μA cm^-2^) and the different concentrations of TMP was evaluated (0.0, 0.03, 0.09, 0.14, 0.27, 0.40, 0.81 and 1.2 μg L^-1^), and the linear response was found between 0.03 and 0.40 μg L^-1^, obtaining the following regression equation: *j* = (-11.6±0.6) *c*_TMP_ + (5.7±0.1), *r^2^* = 0.996 (RSD between 3.0 and 9.5 %). The calibration plot is presented in Figure 4a and representative chronoamperograms are depicted in Figure 4b. The figure of merit results were calculated as described in the literature [34], limits of detection (LOD = 3*S*_x_*/m*) and quantification (LOQ = 10*S*_x_*/m*) were calculated from the calibration straight equation (*S*_x_ corresponds to the standard deviation of the blank and *m* is the slope value), resulting in a LOD 34 ng L^-1^ and LOQ of 115 ng L^-1^.

The precision was assessed (using 0.40 μg L^-1^ of TMP) with three intraday measurements (repeatability), obtaining a coefficient of variation (CV) of 6.1 %, and a CV of 9.3 % for reproducibility (three inter-day evaluations). The coefficient of variation of the method was assessed, obtaining a CV of 8.1 %, which reveals the good precision of the results, as CV <10 %.

The immunosensor response to other important interfering molecules was assessed by analysing ascorbic acid (AA), sulfadiazine (SDZ), and sulfamethoxazole (SMX). The drug selection was based on the possible interference since pharmaceutical compound consumption has led to the release of non-metabolized compounds into the environment, mainly through biological fluids (*e.g.* urine) [2]. Furthermore, the resulting effluents can contaminate water sources and consequently, fishery products [2,4]. To test the biosensor selectivity, the concentration of TMP was kept constant at 0.40 μg L^-1^, and an equal concentration of the selected compounds (0.40 μg L^-1^) was spiked into mackerel samples, which were subsequently analysed. The interference level was expressed as the percentage of TPM in the absence of the tested interference (4.9 %, corresponding to a residual signal background). The results presented in Figure 4c revealed that the other pharmaceutical compounds do not interfere with the TMP analysis since the obtained values varied from 95.5 % for AA, 96.1 % for SDZ and 97.1 % for SMX, which were compared to PBS (100 %), used as a dilution solution. TMP can be co-prescribed with SMX, as TMP-SMX, with the consequent release of these drugs into wastewater, which can increase the quantity detected in fishery products. However, due to the presence of anti-TMP as a bioreceptor in the biosensor construction, high precision (1.5 %) was achieved in the TMP detection, and an efficient electrochemical analysis was performed. The biosensor was also tested with the resuspension solution (PBS) without the pharmaceutical compounds, refuting the low interference level.

### Food sample analysis and method validation

**Figure 5.**
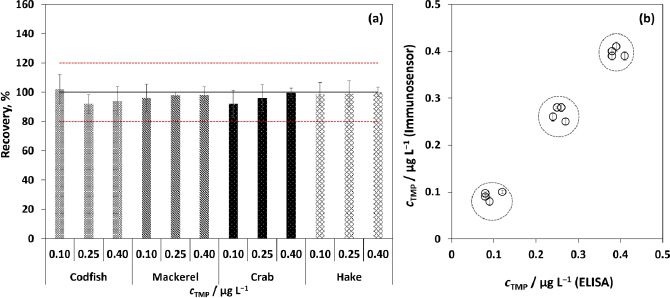
(a) Recovery values obtained for the analysis of codfish, mackerel, crab and hake at distinct TMP concentrations. Red horizontal lines represent the average values ± standard deviation; (b) Correlation values attained using the developed immunosensor and the ELISA

The presence of TMP in food samples was evaluated through recovery experiments. Commercial fishery species of wide local consumption (codfish, mackerel, crab, and hake) were purchased in supermarkets, and the muscle was selected for processing considering its relevance for food safety. The samples were spiked with increasing TMP concentrations (0.10, 0.25 and 0.40 μg L^-1^), and the obtained recoveries varied between 92.0 to 103.2 (codfish), 96.0 to 98.2 % (mackerel), 92.1 to 99.5 % (crab) and 98.7 to 101.1 % (hake), which indicated a negligible matrix effect when compared to the calibration plots obtained under the optimized conditions in PBS. Besides, the results showed that low TMP concentrations can be detected with confidence (RSD between 2.5 to 9.7 %), indicating the potential applicability of the developed biosensor for the analysis of vestigial amounts of the drug in fish tissues, which is particularly important due to the possibility of ingestion in the nourishment (Figure 5a). The biosensor results were compared with those from ELISA and an excellent correlation was reached between the two methods, which validated the developed biosensor since similar quantifiable results were obtained, as expected, for the analysed concentrations (Figure 5b).

The developed competitive-type immunosensor allows accurate analysis of foodstuffs. The amperometric analysis was performed using an innovative 3-electrode configuration and an improved, small-sized electrochemical cell (1×1 cm) suitable for low-quantity samples and rapid analysis. Also, regarding portability, the biosensor can be considered for on-site detection, which is highly advantageous compared with the widely used techniques (*e.g.* liquid chromatography, ELISA). The developed sensor offers key advantages over the gold standard commercial ELISA kits, including a lower detection limit (34 ng L^-1^ *vs.* ~100 to 600 ng L^-1^ for ELISA), shorter assay time (45 min *vs.* 2 to 4 h), and reduced sample volume (200 μL *vs.* 1 to 2 mL). It is also cost-effective and demonstrates high accuracy (92.0 to 103.2 %), reproducibility (CV <10 *%*), and selectivity (bias <5 %), making it suitable for rapid, on-site TMP detection in environmental samples [36]. The main characteristics were compared with those of the existing electrochemical sensors for the analysis of TMP, with most of them being validated in environmental (river, tap and lake water) and pharmaceutical (tablets, urine, and serum) samples (Table 1). Electrochemical sensors utilize the nanostructuring of conventional electrodes, such as GCE [16-20] and CPE [21-26], which are mostly suitable for laboratory work due to their bulkier and conventional three-electrode set-up. In contrast, recent studies have started to explore nanostructured screen-printed electrodes (SPE), reported as an option for tap water, tablets, urine and serum due to the small-sized 3-electrode system and low-volume sample [27,28] or small-sized carbon fibre paper electrodes for the determination of TMP, conferring miniaturisation and portability capacity [29].

**Table 1.** Electrochemical sensor characteristics for TMP analysis, analytical performance and sample application

Sensing surface		Techni-que	Sensor performance			Sample analysis		Ref.
Electrode	Platform modification		Linear range, μg L^-1^	LOD, μg L^-1^	Recovery, %	RSD, %	Application	
GCE	PC-CuPh	SWV	116-319	194	97.4-99.6	<1.0	River water	[16]
	Gr-ZnO	DPV	290-52258	87.1	93.5-103	<3.0	Tap and lake water, urine, serum	[17]
	PC-Chit-EPH-AuNPs	SWV	58-1742	3.6	93-105	<2.7	River water, serum, urine	[18]
	MoO_2_	DPV	580-5806	36.9	89.6-111	-	Tablets	[19]
	GO-ZnO/QDs	SWV	50-300	19.1	-	-	Tablets	[20]
CPE	Graphite/Oil/SiO_2_-GOQDs	DPV	2.9-174	2.6	-	-	-	[21]
	Graphite/Oil/AgNPs	DPV	1.4-145	7.5	92.3-97.1	<4.3	Tap water, urine	[22]
	Graphite/Oil/PS/MWCNT	SWV	58-4064	14.1	100.4-101	<2.3	Tablets, urine, plasma	[23]
	Graphite/Oil/Glucose	DPV	261-29032	6.0	95.4-98.7	<2.7	Tablets, urine, lake water	[24]
	Graphite/Oil/ZnO NPs	DPV	232-2903	7.5	97.5-99.0	<2.1	Tablets, urine	[25]
	(Graphite/Oil/Fe_3_O_4_MWCNT) /rGO/Fe_3_O_4_-MWCNT-MIP	DPV	1.2-23 23-14×10^4^	0.35	91.0-119	<5.0	Tablets, river water, urine	[26]
SPE	GrNR	DPV	290-2903	11.6	97.8-102	<4.0	Tap water	[27]
	ZnO-Tb	AMP	0.29-26×10^4^	0.067	97.8-102	<5.0	Tablets, urine, serum	[28]
CP	-	SWV	14.5-580	18.9	103-107.4	<2.0	Fish (*Merluccius capensis*)	[29]
	Antibody	AMP	0.030-0.40	0.034	92.0-103.2	<5.0	Codfish, mackerel, crab, hake	This work

AgNPs-silver nanoparticles; Chit-chitosan; CPE-carbon paste electrode; CP-carbon paper; CuPh-copper (II) phthalocyanine; EPH-epichlorohydrin; GCE-glassy carbon electrode; GO-graphene oxide; GOQDs-graphene oxide quantum dots; Gr-graphene; GrNR-graphene nanoribbons; MIP-molecularly imprinted polymer; MWCNT-multi-walled carbon nanotubes; NPs-nanoparticles; PC-Printex carbon black; PS-sugar polymer; QDs-quantum dots; rGO-reduced graphene oxide; SPCE-screen-printed carbon electrodes; SPE-screen-printed electrode; AMP-amperometry

From this perspective, the carbon paper electrode was applied as a transducer for the successful TMP analysis in a fish sample (the only previous sensor applied in food samples), obtaining an extremely low LOD of 18.9 μg L^-1^ [29]. However, compared to the present work, a high-volume sample was required since a conventional electrochemical cell was used.

To enhance existing analytical tools, this study introduces an alternative system configuration designed for the selective analysis of TMP. To the best of our knowledge, this is the first electrochemical biosensing strategy utilizing an immobilized biological receptor on paper-based electrodes to quantify TMP in food products.

### Immunosensor′s greenness assessment

**Figure 6.**
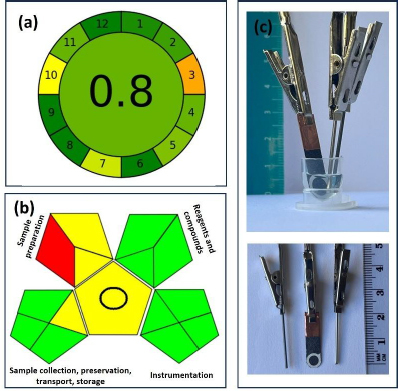
(a) AGREE - Analytical Greenness report (Scores range from 0.0 (red) to 1.0 (dark green)) [35]: 1-Sample treatment (score 0.95), 2-Sample amount (score 0.88), 3-Device positioning (score 0.33), 4-Sample preparation stages (score 0.80), 5-Automation, miniaturization (score 0.75), 6-Derivatization (score 1.0), 7-Waste (score 0.60), 8-Analysis throughput (score 1.0), 9-Energy consumption (score 1.0), 10-Source of reagents (score 0.50), 11-Toxicity (score 0.80), 12-Operator′s safety (score 1.0). Scores range from 0.0 (red) to 1.0 (dark green); (b) GAPI - Green Analytical Procedure Index report [36]: Sample collection, preservation, transport, storage, Sample preparation, Reagents and compounds, and Instrumentation; (c) The cell incorporates a three-electrode setup and has been designed with a specific configuration to support this arrangement

The core innovation of this study lies in the small-sized 3-electrode configuration. This strategy is advantageous compared to paper-based screen-printed electrodes, as no ink or cellulose platform is required for the sensor to operate, eliminating the need for expensive and large-format printers to obtain the electrodes. The green assessment profile was assessed using AGREE - Analytical GREEnness Metric Approach and Software [35]. This tool enables the analytical characterisation of greenness (Figure 6a) and correlates sample preparation, device, automation, waste, analysis throughput, energy consumption, reagents, toxicity, and operator safety. The AGREE score of 0.8 demonstrates the overall sustainability of the sensors, with 10 out of 12 assessed criteria meeting the green profile. Two criteria, device positioning, due to the lack of online analytical capability, and reagent sourcing, given the use of organic solvents in the procedure, require further optimization. In terms of the GAPI assessment (Figure 6b), the method reflects a remarkable green analytical technique with strong environmental compatibility. However, certain limitations remain, particularly concerning the reagents employed during sample preparation. The cell featuring the electrodes and the design of the cell configuration is presented in Figure 6c.

The developed immunosensor offers a promising alternative for rapid, on-site TMP monitoring in fishery species, addressing the limitations of existing methods. Further studies will contribute to understanding the impact of complex sample matrices on sensor performance and optimize sample pre-treatment procedures to enhance accuracy in real-world samples, thereby improving the reliability and applicability of the sensor for routine monitoring.

## Conclusions

As an alternative to the traditional bulky techniques and the previously reported electrochemical sensors for TPM analysis, the present work reports an innovative 3-electrode system (carbon-paper working electrode, pencil graphite lead auxiliary electrode and a stainless-steel needle reference electrode), for rapid (<1 h) analysis of a pharmaceutical compound using a recyclable electrochemical cell of low sample volume (200 μL). A sustainable and innovative carbon paper electrode was used as the WE for the efficient immobilization of the bioreceptor (anti-TMP antibody) and effective target analyte detection. The developed system was applied to the construction of an electrochemical immunosensor, and a competitive-type assay was used for the analysis of fish species. Furthermore, the biosensing approach was assessed through analytical parameters and recoveries were obtained between 92.0 to 103.2 %. The reported tool can be a significant advancement towards the use of biosensors to detect contaminants of emerging concern and food safety and quality control, enabling proactive management of TMP contamination and minimizing its impact on aquatic ecosystems.
